# Efficacy of pre-operative quadriceps strength training on knee-extensor strength before and shortly following total knee arthroplasty: protocol for a randomized, dose-response trial (The QUADX-1 trial)

**DOI:** 10.1186/s13063-017-2366-9

**Published:** 2018-01-18

**Authors:** Rasmus Skov Husted, Anders Troelsen, Kristian Thorborg, Michael Skovdal Rathleff, Henrik Husted, Thomas Bandholm

**Affiliations:** 1 Physical Medicine and Rehabilitation Research - Copenhagen (PMR-C), Copenhagen University Hospital Hvidovre, Hvidovre, Denmark; 2 Department of Physical and Occupational Therapy, Copenhagen University Hospital Hvidovre, Hvidovre, Denmark; 3 Optimed, Clinical Research Centre, Copenhagen University Hospital Hvidovre, Hvidovre, Denmark; 4 Clinical Orthopedic Research Hvidovre (CORH), Department of Orthopedic Surgery, Copenhagen University Hospital Hvidovre, Hvidovre, Denmark; 5 Sports Orthopaedic Research Center – Copenhagen (SORC-C), Department of Orthopedic Surgery, Copenhagen University Hospital Hvidovre, Hvidovre, Denmark; 6 Research Unit for General Practice in Aalborg, Department of Clinical Medicine, Aalborg University, Aalborg, Denmark; 7 Department of Occupational Therapy and Physiotherapy, Aalborg University Hospital, Aalborg, Denmark

**Keywords:** Knee osteoarthritis, Knee-extensor exercise, Exercise therapy, Strength training, Dose-response, Total knee arthroplasty

## Abstract

**Background:**

Patients with knee osteoarthritis (OA) report knee pain, limitation in physical activities and low quality of life. The two primary treatments for knee OA are non-surgical treatment (e.g., exercise) and surgery (total knee arthroplasty (TKA)); however, national guidelines recommend non-surgical treatment to be tried prior to surgical procedures. Patients with knee OA are characterized by decreased muscle strength, particularly in the knee-extensor muscles. Correspondingly, decreased knee-extensor strength is found to be associated with an increased risk of development, progression and severity of knee OA symptoms. Recent trials suggest a positive effect of pre-operative exercise on pre- and post-operative outcome; however, the most effective pre-operative knee-extensor strength exercise dosage is not known. The purpose of the present trial is to investigate the efficacy of three different exercise dosages of pre-operative, home-based, knee-extensor strength exercise on knee-extensor strength before and shortly after surgery in patients eligible for TKA due to end-stage knee OA.

**Methods:**

In this randomized dose-response trial with a three-arm parallel design, 140 patients with end-stage knee OA (candidates for TKA) are randomized to one of three exercise dosages (two, four or six session/week) of knee-extensor strength exercise (three sets, 12 repetitions at 12 RM, per exercise session) for 12 weeks. The knee-extensor strength exercise is home-based (unsupervised) and performed with an elastic exercise band following an initial exercise instruction. Adherence is objectively quantified using a sensor attached to the exercise band. The primary outcome will be the change in knee-extensor strength. Following the 12-week exercise period, the need for TKA surgery is re-assessed by an orthopedic surgeon.

**Discussion:**

Decreased knee-extensor strength is a major challenge in patients with knee OA. Exercise programs focusing on knee-extensor strength are found to be more effective in relieving knee OA pain and symptoms compared to more general exercise programs. However, the optimal exercise dosage for knee-extensor strength deficits in patients with knee OA is inconclusive. Knowledge on the dose-response relationship for knee-extensor strength exercise in patients with knee OA will help guide future non-surgical treatment in this patient population.

**Trial registration:**

ClinicalTrials.gov, ID: NCT02931058. Pre-registered on 10 October 2016.

**Electronic supplementary material:**

The online version of this article (10.1186/s13063-017-2366-9) contains supplementary material, which is available to authorized users.

## Background

Patients diagnosed with knee osteoarthritis (OA) report pain, low quality of life and limitation in physical activities [1]. As a consequence, in Denmark, around 8000 patients with end-stage knee OA receive surgical treatment annually, in the form of total knee arthroplasty (TKA), to overcome their knee-related disabilities [2]. Currently, the two primary treatments for knee OA are non-surgical treatment (e.g., pain treatment, exercise and weight loss if indicated) and surgery (TKA) [3].

There is preliminary evidence that pre-operative exercise may postpone total hip arthroplasty in patients with hip OA [4]. Likewise, pre-operative exercise in candidates for TKA will provide an optimized basis for deciding whether to commence TKA; e.g., patients experiencing pain relief and functional improvement following exercise might benefit from postponing their potential TKA and vice versa. The latest systematic review on the efficacy of pre-operative strength exercise to enhance post-operative recovery after TKA and THA concludes that pre-habilitation may slightly improve early post-operative pain and function among patients undergoing total joint replacement; but the effects are too small and short-lived to be considered clinically important [5]. However, this conclusion is based on trials with significant limitations, providing very low certainty in estimates [5].

Recently, the first randomized controlled trial investigating the efficacy of TKA in patients eligible for TKA was conducted [6]. It showed an added effect (pain relief and functional improvement) of 30% by TKA and non-surgical treatment to that achieved by non-surgical treatment alone (30%). This highlights the importance of coordinating non-surgical and surgical care to select the right candidates for surgery, especially as patients undergoing TKA seem to experience more serious adverse events compared to non-surgical treatment [6]. These results suggest that non-surgical treatment should at least be tried out and considered before commencing surgical procedures. Optimally, patients awaiting TKA would conduct home-based, pre-operative exercise as the effect of exercise helps to establish the potential need for a future orthopedic operation. At the same time, it would be a potential solution of little cost and it enables the patients to self-manage their symptoms.

Patients with knee OA are characterized by decreased knee-extensor strength and this decrease in knee-extensor strength is associated with limitation of activities of daily living, independent of knee pain [7, 8]. Correspondingly, knee-extensor muscle weakness is found to be associated with increased risk of developing knee OA, progression of knee OA, symptoms of knee pain and decline in function [9, 10]. Clinically, patients diagnosed with end-stage knee OA who are awaiting TKA to reduce pain and disability are reported to have 35% reduced knee-extensor strength compared to healthy, age-matched persons [7]. Shortly after TKA, patients lose an additional 80% of their pre-operative knee-extensor strength [11]. This massive loss in knee-extensor strength severely limits functional performance and may delay hospital discharge in the large number of patients undergoing TKA every year [12]. One of the latest systematic reviews on the efficacy of exercise to reduce pain and disability in patients with knee OA showed that isolated knee-extensor strength exercise was more effective in reducing pain and disability if not combined with other forms of exercises (e.g., other muscle groups or cardiovascular training) [13]. In line with this, the 2014 international guidelines for non-surgical management of knee OA include strength exercise of the knee-extensor muscles [14].

Two recent randomized controlled trials have indicated that high-volume, pre-operative strength exercise may enhance pre- and post-operative knee-extensor strength as well as functional performance in patients undergoing TKA [15, 16]. Hence, there are indications that the applied pre-operative exercise dose seems related to pre- and post-operative efficacy, making a dose-response trial of pre-operative exercise particularly relevant.

### Purpose

The purpose of the present trial is to investigate the efficacy of three different exercise dosages (two, four and six exercise sessions per week) of pre-operative, home-based, knee-extensor strength exercise on knee-extensor strength before and shortly after surgery in patients eligible for TKA due to end-stage knee OA.

### Hypothesis

We hypothesize that a dosage of four knee-extensor strength exercise sessions per week will elicit the greatest strength increase pre-operatively compared to two or six sessions per week. The recommended minimum exercise dosage required for strength gains according the American College of Sports Medicine is two sessions per week [17]; therefore, two greater dosages are investigated and used as comparators. Four sessions per week is likely optimal and six sessions per week probably have no additional benefit, but could potentially increase knee pain [18, 19].

## Methods

### Literature search and search matrix

Before commencing the planning of this trial, a systematic literature search was conducted to locate trials investigating the same research question or planning to do so (protocols). The following search matrix (developed using a PICOT approach [20]) was used to search MedLine through pubmed.com on 18 November 2015 with weekly updated searches:

(((((((((((“end stage osteoarthritis”) *or* ((“Osteoarthritis”[Mesh]) *or* “Osteoarthritis, Knee”[Mesh])) *or* osteoarthritis)))) *or* “knee osteoarthritis”[Title/Abstract])) *and* (((((((((“resistance training”[Title/Abstract]) *or* “Resistance Training”[Mesh]) *or* “strength training”[Title/Abstract]) *or* physiotherapy[Title/Abstract]) *or* “Physical Therapy Specialty”[Mesh]) *or* “Physical Therapy Modalities”[Mesh]) *or* “knee extensor training”[Title/Abstract]) *or* “quadriceps training”[Title/Abstract]) *or* “pre-operative training”[Title/Abstract]) *or* “physical function”[Title/Abstract])) *and* ((((((“pre-operative strength”[Title/Abstract]) *or* “knee extensor strength”[Title/Abstract]) *or* “quadriceps strength”[Title/Abstract]) *or* “Pain”[Mesh]) *or* pain[Title/Abstract]) *or* “Musculoskeletal Pain”[Mesh])) *and* (((((((“post-operative strength”[Title/Abstract]) *or* “post-operative knee extensor strength”[Title/Abstract]) *or* “knee extensor strength”[Title/Abstract]) *or* “quadriceps strength”[Title/Abstract]) *or* “Pain”[Mesh]) *or* pain[Title/Abstract]) *or* “Musculoskeletal Pain”[Mesh]) *and* (((((((meta-analysis[Title/Abstract]) *or* “Meta-Analysis”[Publication Type]) *or* “systematic review”[Title/Abstract]) *or* “Review”[Publication Type]) *or* “randomized controlled trial”[Title/Abstract]) *or* “Randomized Controlled Trial”[Publication Type]) *or* “prospective cohort”[Title/Abstract]).

Systematic reviews and meta-analyses were found but none specifically addressed the pre-operative effect on muscle strength of a single knee-extensor strength exercise in patients with knee OA. No Cochrane reviews were found. An updated search was performed on 1 May 2017 providing new academic literature relevant for the scope of this trial [21, 22].

### Trial design

**Fig. 1:**
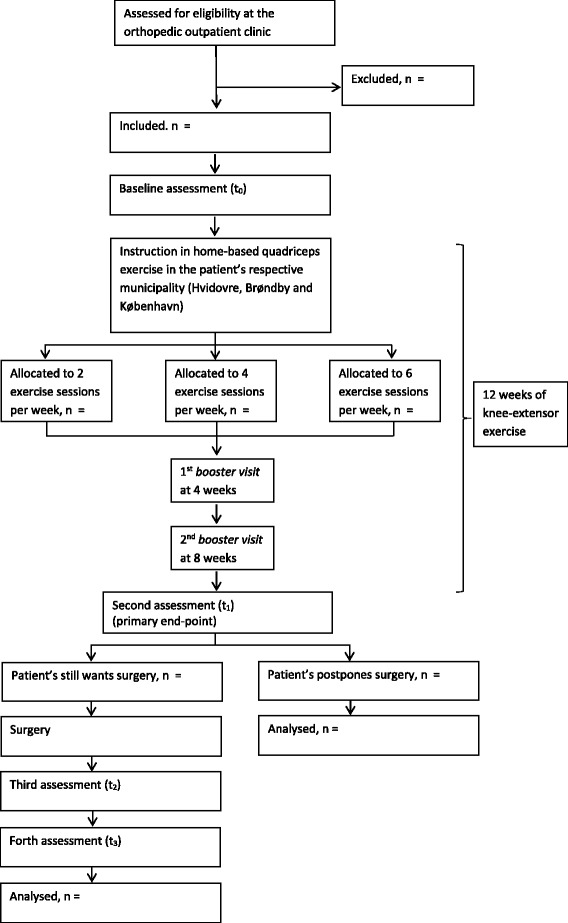
Flow chart of enrollment, randomization, treatment and follow-up

The trial is named the *QUADX-1 trial*. It uses a three-arm, parallel-group, randomized trial design with three intervention groups (exercise dosages) and no control group. No control group is included as the primary purpose of the trial is to investigate the dose-response relationship of the investigated knee-extensor strength exercise dosages. Based on the sample size estimation outlined below, 140 patients will be randomized (1:1:1) to one of three exercise dosages for 12 weeks. Outcomes will be assessed blinded at baseline before the start of the exercise (t_0_), after the exercise intervention, which is before possible surgery (t_1_, primary endpoint), at hospital discharge 1–8 days after surgery (if performed) (t_2_) and, finally, at 3 months after surgery (if performed) (t_3_). A flow chart of the trial is provided below (Fig. 1).

**Fig. 2:**
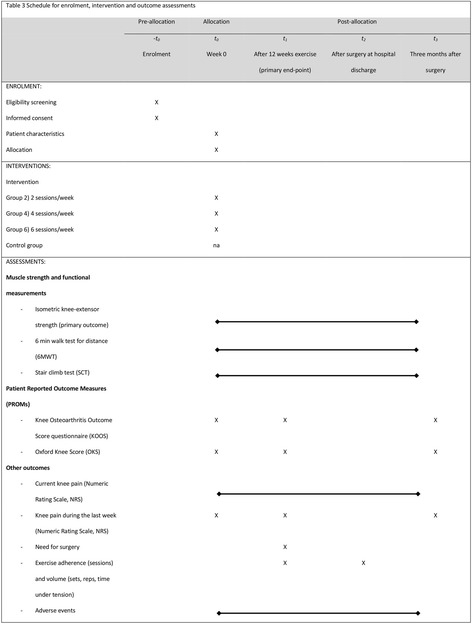
Schedule for enrollment, intervention and outcome assessments (Standard Protocol Items: Recommendations for Interventional Trials (SPIRIT))

This clinical trial protocol is based on the PREPARE trial guide [23] and the SPIRIT Checklist (Fig. 2, Additional file 1) [24]. The trial report will adhere to the Consolidated Standards of Reporting Trials (CONSORT) Checklist using the extension for non-pharmacological treatments [25]. The TIDieR Checklist will be used for intervention description (Additional file 2) [26]. The trial was pre-registered at ClinicalTrials.gov on 10 October 2016 (ID: NCT02931058, https://clinicaltrials.gov/ct2/show/NCT02931058) and approvals with the Ethics Committee of the Capital Region Denmark (ID: H-16025136) and the Danish Data Protection Agency (J. nr.: 2012-58-0004. Lokale RegH j. nr.: AHH-2016-072, med I-Suite nr.: 04980) were obtained before the first patient was enrolled. The trial will be conducted at Copenhagen University Hospital Hvidovre and in the collaborating municipalities of Copenhagen, Hvidovre and Brøndby, Denmark.

### Recruitment

All patients will be included by consecutive sampling from the orthopedic outpatient clinic at Copenhagen University Hospital, Hvidovre. At the orthopedic outpatient clinic, possible patients for TKA surgery due to end-stage knee OA are introduced to the trial and offered to participate by the orthopedic surgeon if they fit the initial eligibility criteria (please see below). On the same day, eligible patients interested in participating in the trial are then referred to a research assistant who provides thorough oral and written information about the trial. Eligible patients are informed that participation in the project includes 12 weeks of home-based, knee-extensor strength exercise with the purpose of improving knee-extensor strength, relieving knee pain and improving functional performance. Further, they are informed that after the exercise period, they will be re-assessed at the orthopedic outpatient clinic with regards to undergo surgery or not. To prevent performance bias, eligible patients are blinded to the exercise dosage randomization as well as the trial hypothesis. Information about the trial is provided in a calm setting dedicated to the trial. Should the patients wish for a companion to be present during the information meeting a new information appointment is scheduled. Along with the information, a second more thorough screening of inclusion and exclusion criteria is commenced. The patients are given a minimum of 24 h to decide whether they would like to participate in the trial or not. If the patients decide to participate, a baseline assessment is scheduled. Written informed consent is obtained at the baseline assessment. Once written informed consent and baseline assessment is completed, the patient is fully included in the trial. Outcome assessments will be performed blinded by the primary investigator and a research assistant dedicated to the trial, who are both trained according to the trial outcome-assessment protocol to ensure standardized assessments throughout the trial. To further refine and ensure standardization of the trial outcome-assessment protocol we piloted the outcome-assessment procedures in seven patients prior to initiating the trial.

### Eligible criteria

#### Inclusion criteria

- Patient is a possible candidate for a primary TKA due to knee OA, based on the below terms:
  - ◦ Knee pain ≥ 3 (Numeric Rating Scale) in the last week
  - ◦ Kellgren-Lawrence classification grade ≥ 2
- Patient is eligible for home-based, knee-extensor strength exercise
- Patient is age ≥ 45 years
- Patient is resident in one of the three municipalities (København, Hvidovre or Brøndby) involved in the trial
- Patient is able to speak and understand Danish

#### Exclusion criteria

- Exercise is contra-indicated for the patient
- Patient has a neurological disorder
- Patient has a diagnosed systemic disease (American Society of Anesthesiologists’ physical status classification score (ASA) ≥ 4)
- Patient with terminal illness
- Patient has severe bone deformity demanding use of non-standard implants
- Weekly alcohol consumption above national recommendations (>7 units for women, > 14 units for men)

#### Protocol amendments

- 8 February 2017: Inclusion criteria *age* changed from *≥ 50 years* to *≥ 45 years*. The rationale for this modification is that patients aged 45–49 years with knee OA who are possible candidates for TKA are likely to benefit from participation. Recent comparable trials of pre-operative exercise on the same population had age-related inclusion criteria including the age-span of 45–49 years of age [27, 28]
- 8 February 2017: Exclusion criteria *ASA score* changed from *≥ 3* to *≥ 4*. The rationale for changing the exclusion criteria relating to the ASA score is that patients with knee OA and an ASA score of 3 who are possible candidates for TKA are likely to benefit from participating in the trial. An ASA score of 3 is defined as “*A patient with severe systemic disease*,” while an ASA score of 4 is defined as “*A patient with severe systemic disease that is a constant threat to life*” [29]. One definition-difference between ASA score 3 and 4 is the functional capacity, where a patient with an ASA score of 3 is able to complete a flight of stairs or walk 200 m on level ground, whereas a patient with an ASA score of 4 is not able to do this [29]. Thus, patients with knee OA and an ASA score of 3 who are possible candidates for TKA are able to participate in the present exercise trial
- 30 March 2017: Inclusion criteria *knee pain (Numeric Rating Scale (NRS)) in the last week* changed from *> 3* to *≥ 3*. The rational for changing the NRS from > 3 to ≥ 3 is that some patients who are considered candidates for total knee replacement might not have pain scores higher than 3. Thus, they might end up being excluded from the trial even though they are clinically considered candidates for total knee replacement. Hence, this change was made to reflect current clinical practice
- 30 March 2017: Inclusion criteria *Oxford Knee Score* (OKS) was removed as an inclusion criterion but still kept as a descriptive and effect outcome. The rationale for this modification (removing OKS as an inclusion criteria) is that OKS is not used in daily clinic practice as a means of assessing whether a patient is a candidate for TKA or not. Thus, keeping the OKS as an inclusion criterion in the QUADX-1 trial will not reflect current clinical practice
- All the above protocol amendments are approved by the Ethics Committee of the Capitol Region Denmark (ID: H-16025136; 55528, 55529, 57312)

### Intervention

Once the baseline assessment is completed, the patients are referred to their local municipality rehabilitation setting for knee-extensor strength exercise instruction. The initial exercise instruction takes place there, where the patients are received by a trial-dedicated physiotherapist who is specialized in instructing and training patients with knee OA. Once the instruction is completed and the patients are comfortable with the exercise, an exercise session is completed under supervision from the physiotherapist. The patients are handed personal elastic exercise bands for exercise at home and a study-specific brochure where instruction notes to the exercise are illustrated and described (Additional file 3). All the physiotherapists dedicated to the trial were trained by the primary investigator prior to the start of the trial to ensure standardized exercise instruction and information across the physiotherapists.

The intervention period is 12 weeks. After 4 and 8 weeks of exercise, the patients have an exercise quality check-up (booster visit) with the physiotherapist in the municipality setting. The exercise quality check-up includes exercise technique re-assessment (fractional and temporal distribution of the contraction modes, range of motion and positioning), ensuring optimal length or type of elastic exercise band (resistance corresponding to 12 repetitions-maximum (RM)) and exercise-related questions from the patients.

Three exercise dosages are investigated; two, four and six sessions per week for 12 weeks (group 2, 4 and 6, respectively) (Table 1). Each exercise session comprises a single-strength training exercise, knee-extension, which is performed in three sets with 12 repetitions at a load corresponding to 12 RM in each set. There is no control group. The patients are randomly allocated to one of the three exercise dosages. The patients are instructed to perform only one exercise session per day. That is, they are not allowed to combine several exercise sessions on the same day. For example, patients randomized to group 6 are instructed to exercise 6 days of the week.

**Table 1:** Exercise sessions per week according to exercise dosage randomization

Dosage groups	Sessions/week					
Gp. 2 – 2 sessions/week	3*12 RM			3*12 RM		
Gp. 4 – 4 sessions/week	3*12 RM	3*12 RM		3*12 RM	3*12 RM	
Gp. 6 – 6 sessions/week	3*12 RM	3*12 RM	3*12 RM	3*12 RM	3*12 RM	3*12 RM

Knee-extensor exercise dosages investigated

**Fig. 3:**
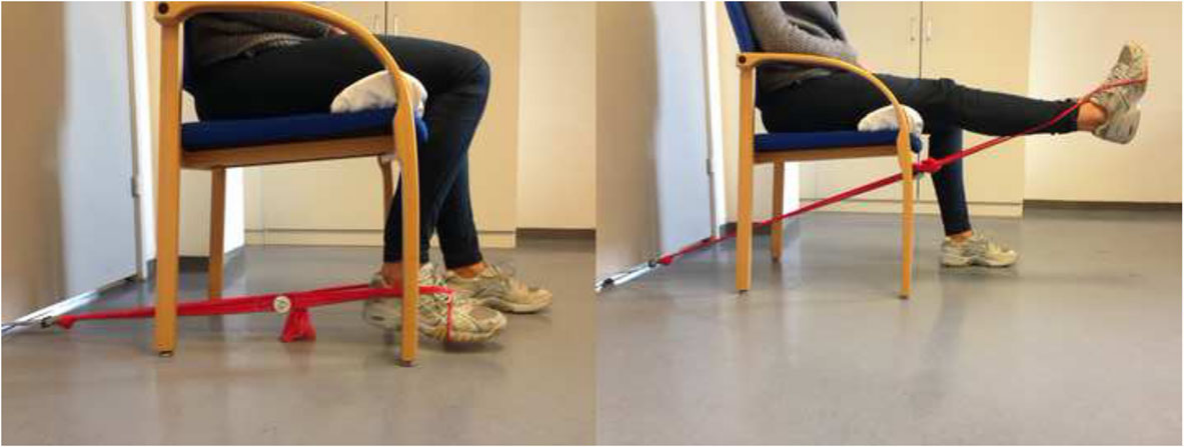
The home-based, knee-extensor strength exercise

The intervention is personalized to the extent where each patient is exercising with an individual absolute resistance in the elastic exercise band corresponding to a relative load of 12 RM. Contractions should be continued until volitional muscular failure. That is, until the knee-extensor muscles are maximally fatigued and the patient is not able to perform further repetitions. If volitional muscular failure occurs before 12 RM, the resistance of the elastic band is adjusted so that the pre-determined number of repetitions can be completed. Whenever a given resistance in the elastic exercise band becomes too low (i.e., more than 12 repetitions per set can be performed), the patients are instructed to adjust the resistance in the elastic exercise band (increase the distance between the two endpoints of the elastic exercise band, i.e., moving the chair further away from the door (Fig. 3)) to achieve a new resistance corresponding to a relative load of 12 RM. The home-based, knee-extensor strength exercise (Fig. 3 and Fig. 9, Supplementary online video [30]) is described in detail below (Table 2) according to the mechano-biological descriptors from Toigo and Boutellier [31].

**Table 2:** Exercise description

Brief name	Home-based, knee-extensor strength exercise Detailed and practical demonstration of the exercise can be accessed via this online video [30]
1. Load magnitude	Corresponding to 12 repetitions maximum (RM)
2. Number of repetitions	12
3. Number of sets	3
4. Rest between sets	Minimum 30 s, or until sufficiently recovered from previous set
5. Number of exercise interventions	Group 2 (2 sessions per week) Group 4 (4 sessions per week) Group 6 (6 sessions per week)
6. Duration of the experimental period	12 weeks
7. Fractional and temporal distribution of the contraction modes per repetition and duration (s) of 1 repetition	Concentric phase (3 s) Isometric phase (1 s) Eccentric phase (4 s) Total duration of 1 repetition (8 s)
8. Rest between repetitions	None, that is, right after finishing one repetition the next is commenced
9. Time-under-tension (TUT)	Repetition TUT (8 s) Set TUT with 12 repetitions (1 min, 36 s) Session TUT (3 sets × 12 repetitions) (4 min, 42 s)
10. Volitional muscular failure	No, but contractions should be continued until volitional muscular failure is very close. That is, until the knee-extensor muscles are maximally fatigued and the patient is not able to perform further repetitions. If volitional muscular failure occurs before the 12 RM, the resistance of the elastic band is adjusted so that the pre-determined number of repetitions can be completed
11. Range of motion	Starting position: 80–90° of knee flexion (0 = full extension) End-range of motion position: 0–10 (as close to full extension as possible)
12. Recovery time between exercise sessions	Group 2: 3 days Group 4: 1 day Group 6: 0 days
13. Anatomical definition of the exercise (exercise form)	The knee-extensor strength exercise is performed sitting in a stable chair. Sitting position in the chair is determined by the distance from the edge of the seat to the back of the knee; this distance should be 2–3 cm. If possible, the back rest can be used as well as the armrest. If the chair is without an armrest one can hold at the (side) edge of the seat. To ensure that the foot is free of the floor at 80–90° of flexion an object (e.g., a pillow) is placed under the thigh (Fig. 3). The elastic exercise band is fixated to an immoveable object (e.g., an elastic exercise band anchor behind a closed door) and wrapped around the ankle of the exercised leg (Fig. 9). Starting position: The exercise leg is relaxed with 80–90° of flexion (0 = full extension) Concentric phase: The exercise leg is extended as much as possible towards full extension using 3 s Isometric phase: The extended position is held for 1 s Eccentric phase: Slow controlled flexion of the knee joint (4 s) until the knee joint is flexed 80–90° These slow movements are chosen to ensure long time-under-tension (TUT) in the muscle

The home-based, knee-extensor strength exercise described in detail according to the mechano-biological descriptors from Toigo and Boutellier [31]

### Criteria for modifying and discontinuing

#### Criteria for discontinuing

Patients are instructed to stop exercising if they experience a *strong flare up* or a *strong aggravation* of knee-related symptoms (e.g., pain and swelling), i.e., knee-related symptoms that are intolerable for the patient. Correspondingly, patients are encouraged to complete the exercise if they experience minor and moderate knee-related symptoms [32]. Should the patients experience intolerable symptoms (stopping with exercise); they are provided with a telephone number to a physiotherapist (the trial manager) and are encouraged to call. The trial manager fills out a standardized form at these calls.

#### Criteria for modifying

Should the patients experience strong knee-related symptoms, they are instructed to lessen the resistance in the elastic exercise band (shortening the distance between the endpoints of the elastic exercise band, i.e., moving the chair closer to the door (Fig. 3)). Importantly though is that this reduced resistance in the elastic exercise band does not comprise the exercise resistance corresponding to 12 RM, (i.e., too little resistance in the elastic exercise band). This is explained to the patients at the exercise instruction session and mentioned in the brochure handed to the patients along with the elastic exercise band (Additional file 3).

### Participant compliance, retention and concomitant care

At the baseline outcome assessment and at the exercise instruction session the patients are encouraged to complete the full intervention and the patients are handed an information brochure about both how to conduct the exercise, with encouragement to complete the intervention and with information on how to handle kinesiophobia (Additional file 3). Further, the patients have two exercise quality check-up visits (booster visits): one at 4 weeks and one at 8 weeks, with the physiotherapist in the municipality setting. These booster visits also serve as promoters for exercise adherence. The patients should continue their lives without changing any concomitant care or interventions, except extra exercise of the quadriceps muscle. For pain relief the patients are allowed use of non-steroidal anti-inflammatory drugs (NSAIDs) and other pain-reducing products (cf. the Danish National Guidelines for knee OA [3]), as they would normally do, not needing a physician’s prescription.

### Outcomes

The trial is designed with four pre-determined outcome assessments; at baseline (week 0) (t_0_), after 12 weeks of home-based exercise (2–8 days after the final exercise session), corresponding to the endpoint before surgery (t_1_), at hospital discharge 1–8 days after surgery (t_2_) (provided surgery is performed) and finally, 3 months after surgery (t_3_) (Fig. 2) (provided surgery is performed). The primary endpoint is after the intervention period (t_1_). Secondary endpoints of interest are just before hospital discharge 1–8 days after surgery (t_2_) and 3 months after surgery (t_3_).

#### Primary outcome

Change in isometric knee-extensor strength from baseline (week 0) to after the intervention period (> 12 weeks). Knee-extensor strength is chosen as the primary outcome for the following reasons. Firstly, it is an outcome closely related to the exposure (strength training), which we consider important in a dose-response trial, because other health effects are likely mediated via increased knee-extensor strength. Secondly, knee-extensor strength is associated with the development and progression of knee OA and knee pain and function [9, 10], and as such, we also consider the outcome, a surrogate measure for the development and progression of knee OA, knee pain and function.

#### Secondary outcomes

Change in performance-based function (walking distance in 6 min and climbing of stairs), current knee pain and during the last week (numerical rating scale), self-reported disability (Knee Osteoarthritis Outcome Score and Oxford Knee Score) as recommended by Osteoarthritis Research Society International [33, 34], need for surgery and exercise adherence (sessions, sets, reps, TUT).

#### Other outcomes

Registration of adverse events (adverse event, number of adverse events in each group (surgery/no surgery).

At outcome assessment endpoint t_2_ (after surgery at hospital discharge) only the outcomes *isometric knee-extensor strength, 6-Minute Walking Test (6MWT), SCT* and *current knee pain* are assessed. The *KOOS* and *OKS* questionnaires as well as *knee pain during the last week* are omitted at this endpoint as they are not validated to assess acute post-operative conditions, and use too long a recall period.

### Elaborated description of outcome measures

#### Primary outcome

##### Isometric knee-extensor strength

The measurement will be assessed using a computerized strength chair (Good Strength Chair, Metitur Oy, Jyvaskyla, Finland). This is a valid (0.78–0.92) and reliable (inter-trial 0.98–1.00 (standard error of measurement (SEM) < 10%), inter-evaluator 0.92–0.99 (SEM 8.34–9.92%)) knee-extensor strength measure in the TKA population [35].

Prior to outcome assessment, the patients will be informed about the procedure. The measurement consists of five maximal isometric knee-extensor contractions at 60° knee flexion separated by a 60-s pauses. The highest obtained value will be used for analysis. The patients are instructed to extend their knee as forcefully as possible with a gradual increase in force over a 5-s period. There will be strong and standardized verbal encouragement during each contraction. Knee-extensor strength will subsequently be expressed as the maximal voluntary torque per kilogram body mass (Nm/kg) using the external lever arm and body mass of each patient. Results will be presented, firstly, as absolute changes (Nm/kg) and, secondly, as relative changes (%) from baseline.

#### Secondary outcomes

##### 6-Minute Walking Test for distance (6MWT)

The 6MWT measures the maximal distance a patient is able to walk in 6 min between two cones placed 29 m apart from each other. The measurement has previously been found to be reliable (intraclass correlation coefficient (ICC)_2,1_ 0.97, SEM 13.0 m) [36] and responsive [37] in the TKA population.

The patients are instructed to walk as long a distance as they can in six minutes. They will be encouraged to walk as fast as possible but are not allowed to jog or run. The patients are allowed to rest standing or leaning against a wall during the six minutes but the time will be running. Walking aids are allowed if needed. At each minute the patients will be informed of the time.

##### Stair Climb Test (SCT)

The SCT measures the time (seconds) it takes to ascend and descend an 11-step flight of stairs with 16-cm step height. The stair has a handrail on both sides. The SCT has been found to be reliable in the TKA population (inter-tester ICC_2,1_ 0.94–0.96, SEM 1.14 s, minimal detectable change (MDC)_90_ 2.6 s) [38]. The patients are instructed to ascend and descend an 11-step flight of stairs as fast as possible, but in a safe manner. Use of hand rail and walking aid is permitted. The patients are allowed to rest during the measurement but the time keeps running.

##### Knee Osteoarthritis Outcome Score (KOOS)

The KOOS is a 42-item questionnaire regarding knee function. The questionnaire is comprised of five subscales (symptoms (7), pain (9), function, daily living (17), function, sports and recreational activities (5) and quality of life (4). Each question has standardized answer options with five options at each question (Likert scale, 0–4). After normalization of the answers each subscale scores from 0–100 (100 indicating no symptoms). The KOOS questionnaire is found to be reliable in all subscales (pain ICC 0.8–0.97, SEM 7.2–10.2; symptoms ICC 0.74–0.94, SEM 7.2–9.0; daily living ICC 0.84–0.94, SEM 5.2–11.7; sports ICC 0.65–0.92, SEM 9–24.6; quality of life ICC 0.6–0.91, SEM 7.4–10.8) [39]. The KOOS questionnaire is also found be to reliable in the TKA population [40].

##### Oxford Knee Score (OKS)

The OKS is a 12-item questionnaire regarding knee-related function and pain in patients with knee OA. Each question has five answer options (Likert scale, 0–4). The OKS demonstrates good test-retest reliability for both the summary scale (ICC 0.93, MDC_90_ + 6), pain (ICC 0.91, MDC_90_ ± 16) and function (ICC 0.92, MDC_90_ ± 15) component subscales [41].

##### Knee pain

Individual knee pain is assessed with the Numeric Rating Scale (NRS). This is an 11-point subjective pain scale ranging from 0–10 (0 indicating no pain). In this trial, the patients will be asked about their knee pain related to two endpoints: (1) knee pain right now and (2) during the last week. The question is asked in the following manner “on a scale from 0 to 10 where 0 indicates no pain and 10 indicates worst imaginable pain, how much knee pain do you have (1) right now and (2) how much knee pain have you had in the last week (index knee)?” The patients are asked while seated in a chair with 70–90° of knee flexion (standardized). The NRS is found to have the strongest face validity compared to other pain measurement scales (Visual Analog Scale and Verbal Descriptor Scale) in surgical patients after surgery as well as high construct and criterion validity [42]. The NRS is also found to be reliable both before (ICC 0.82) and in the first 1–6 days following surgery (ICC 0.673–0.783) [43]. A minimal clinically important difference in pain relief post orthopedic surgery has previously been suggested to be 35% [44].

##### Surgical status – Need for surgery?

At the second outcome assessment (after the 12-week exercise period (t_1_)) the patients are asked by the outcome assessor “*based on your knee symptoms in the last week would you say that you need knee surgery?*” The outcome assessor is a physiotherapist with insight to the knee OA condition. The answer will be categorized into one of three options: (1) “*yes*” *I believe I need surgery*, (2) *I do not know* or (3) “*no*” *I do not believe I need surgery*.

##### Exercise adherence

A large challenge regarding home-based exercise is that adherence to home-based exercise is reported to be poor [45–48], suggesting low effect of the exercise interventions. To take into account the possibility of non-adherence to the intervention (which could distort the possible conclusion that the intervention did not work), we will objectively quantify exercise adherence. Adherence to the home-based, single knee-extensor strength exercise will be assessed using a sensor (BandCizer technology) attached to the elastic exercise band used for the knee-extensor strength exercise. The sensor stores data on date, time, number of sets, repetitions, tonnage (kg × repetitions) and TUT. This elastic exercise band sensor technology has been reported to be valid [49] and reliable (ICC > 0.99) [50] for quantification of total repetition, single repetition and contraction specific TUT of an unsupervised exercise intervention.

**Fig. 4:**
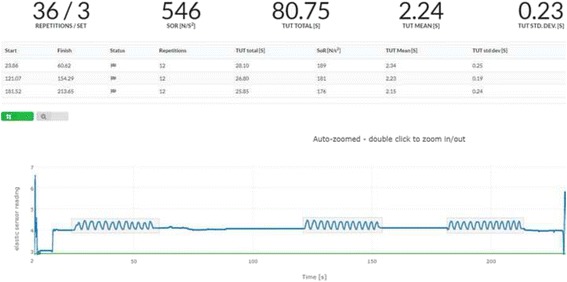
Objective quantification of exercise adherence for a full exercise session comprising three sets of 12 repetitions

**Fig. 5:**
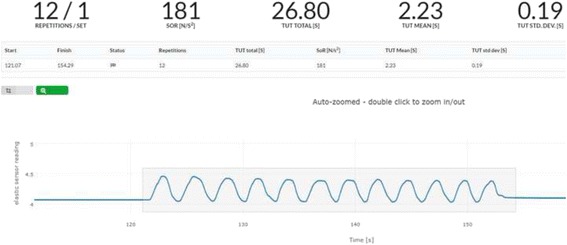
Objective quantification of exercise adherence for a single exercise set of 12 repetitions

In the present trial, patients are defined as adherent to the exercise intervention if > 75% of the prescribed exercise sessions are completed. Correspondingly, 1.5 sessions/week must be completed in group 2, three sessions/week in group 4 and 4.5 sessions/week in group 6. After the 12-week exercise period, the patients who undergo surgery are encouraged to continue exercising (same dosage) until the day of surgery. The exercise adherence during this period will also be quantified by use of the sensor. Patients deciding not to undergo surgery following the 12 weeks of exercise are encouraged to keep exercising, but exercise adherence will not be quantified. Figure 4 shows an example of objectively quantified exercise adherence via the sensor attached to the elastic exercise band. The example shows a knee-extensor strength exercise session composed of three sets with 12 repetitions in each set resulting in 36 repetitions in total for the exercise session. Number of repetitions, total TUT, mean TUT for the 36 repetition and the corresponding standard deviation can be extracted. This is also possible for single exercise sets (Fig. 5).

##### Adverse events

All adverse events occurring while the patients are enrolled in the trial will be recorded regardless of their relation to the exercise intervention, surgery or occurrence likely not related to the trial. Thus, an adverse event can both be a negative effect of the intervention, surgery or an occurrence not related to the trial, that is an untoward occurrence during the trial which may or may not causally related to the intervention or trial. Regardless of a relation to the trial, all adverse events are recorded and reported.

Finally, a qualitative study will be embedded in the trial. Semi-structured interviews will be performed with randomly selected participating patients both before and after the trial about their experienced enablers and barriers related to the knee-extensor strength exercise and adherence to the home-based intervention. The orthopedic surgeons allocated to the trial will also undergo semi-structured interviews about their experienced enablers and barriers with the non-surgical, pre-operative, home-based intervention both before and after the trial. The physiotherapists allocated to the trial will undergo focus interviews, both before the trial is commenced and once the trial is completed, to explore their experienced enablers and barriers related to administering the home-based intervention. This embedded qualitative study is undertaken to refine the home-based intervention for future trials and clinical implementation. This knowledge can then be used to design a context-specific implementation plan, given a positive trial outcome. The embedded qualitative study will be reported in a separate qualitative paper, with a clear reference to the QUADX-1 trial.

Regular auditing is planned during the trial. Regular meetings between the primary investigator, the orthopedic department, the municipalities and the research team will allow for checking of treatment notes and outcome assessment forms for fidelity to protocol which allows for addressing deviations.

### Sample size

For a three-group-level One-way ANOVA of a normal mean difference with a two-sided significance level of 0.05, a common standard deviation of 0.22 Nm/kg (isometric knee-extensor strength measurement) [51], and a minimal clinically important difference of 0.15 Nm/kg (15%), a sample size of 126 (42 per group) patients is required to obtain a power of 80%. To allow for a dropout rate of 10%, we will include 140 patients in total for the intention-to-treat (ITT) analysis (3 × 42 + 14 = 140).

### Randomization

The patients will be randomly assigned to one of the three intervention groups (two, four or six sessions per week) by a 1:1:1 allocation ratio. The generation of the allocation sequence will be attended to by a statistician not involved in the trial in any other way. One hundred and forty opaque and sealed envelopes will be generated. After being included in the trial (signed written informed consent and completion of baseline assessment) a person independent of the trial will open one of these envelopes and inform the patient’s municipality which of the three exercise groups the patient is allocated to. In this way, the allocation information is kept secret from the outcome assessor.

### Blinding

The primary investigator collecting the outcomes (outcome assessor) as well as the data analysts will be blinded to allocation. At outcome assessment sessions, the outcome assessor will start by informing the patients that they are not allowed to mention what exercise dosage they have received. The data will be coded in such a way that group allocation is concealed in the dataset, thus blinding the outcome assessors and data analysts to the group allocation. The patients and the physiotherapists instructing in the intervention will not be blinded due to the nature of the intervention; however, the patients will be blinded to the exercise dosages in the other groups, which exercise dosages will be compared in the analysis and which dosage is hypothesized to have the largest effect. Unblinding will only happen in the case where the wellbeing a the patient is at risk. This will be assessed in collaboration with the patient’s physician.

### Data management

Data from the isometric knee-extensor strength assessment are stored on a computer dedicated to the Metitur equipment as well as being recorded in handwriting in a standardized Case Report Form. The self-reported questionnaires (KOOS and OKS) will be filled in by the patients in paper formats, as this is the way these questionnaires are designed to be filled in. All data from the functional (6MWT and SCT) and pain (NRS) assessments will be recorded on a standardized Case Report Form by the outcome assessor.

Data on exercise adherence from the sensors is saved continuously on the built-in SD card. After the intervention period, the sensors are collected and the exercise data are transferred by Bluetooth from the SD card onto a secure server. The device will not contain any personal data.

Following all outcome assessments, data will be entered into the browser-based research database Research Electronic Data Capture (RedCap 7.1.1) by trial personal using blinded double-data entry to ensure data quality. To further ensure the integrity of the data, anonymous ID numbers will be applied and data quality, data range and data values will be checked to minimize typing errors. All original written information and case report forms will be stored in a secure locker and saved for 3 after the completion of the trial at the trial location. All electronic data will be anonymous (patient IDs), coded and saved on a secure server in the Capital Region of Denmark.

### Statistical analysis

#### Analysis outline

Three groups; group 2 = two sessions/week, group 4 = four sessions/week and group 6 = six sessions/week. There is no control group. The primary outcome is the change in isometric knee-extensor strength. Time from baseline to after 12 weeks of exercise is the primary endpoint (Δ_t0-t1_) and time from baseline to just before hospital discharge, and time from baseline to 3 months after surgery are the secondary endpoints (Δ_t0-t2_ and Δ_t0-t3_, respectively). The analysis plans for the primary and secondary analyses are outlined in Table 3.

**Table 3:** Analysis outline for primary and secondary analysis

Variable/outcome	Hypothesis	Outcome measure (unit, scale)	Methods of analysis
Descriptive statistics (sample characteristics)		Age, weight, height, side of index knee (continuous and dichotomous)	Summary statistics
Primary analysis			
Primary outcome			
1. Change in *isometric knee-extensor strength* Δ_t0-t1_	Group 2 < Group 4 Group 4 ≈ Group 6	Change in Nm/kg (continuous)	Analysis of variance ANOVA^a^
Secondary analysis			
Secondary outcomes			
2. Change in *isometric knee-extensor strength* Δ_t0-t2_, Δ_t0-t3_	Group 2 < Group 4 Group 4 ≈ Group 6	Change in Nm/kg (continuous)	ANOVA^a^
3. Change in *Knee Osteoarthritis Outcome Score* (KOOS) subscales Δ_t0-t1_, Δ_t0-t3_	Group 2 < Group 4 Group 4 > Group 6	Change in questionnaire subscales (continuous)	ANOVA^a^
4. Change in *Oxford Knee Score* (OKS) Δ_t0-t1_, Δ_t0-t3_	Group 2 < Group 4 Group 4 > Group 6	Change in questionnaire (continuous)	ANOVA^a^
5. Change in *6-Minute Walking Test for distance* (6MWT) Δ_t0-t1_, Δ_t0-t2_, Δ_t0-t3_	Group 2 < Group 4 Group 4 > Group 6	Change in meters walked (continuous)	ANOVA^a^
6. Change in *Stair Climb Test* (SCT) Δ_t0-t1_, Δ_t0-t2_, Δ_t0-t3_	Group 2 < Group 4 Group 4 > Group 6	Change in time used to ascend and descend stairs (continuous)	ANOVA^a^
7. Change in *current knee pain* (Numeric Rank Scale, NRS) Δ_t0-t1_, Δ_t0-t2_, Δ_t0-t3_	Group 2 < Group 4 Group 4 > Group 6	Change in NRS 0–10 (continuous)	ANOVA^a^
8. Change in *knee pain during the last week* (Numeric Rank Scale, NRS) Δ_t0-t1_, Δ_t0-t3_	Group 2 < Group 4 Group 4 > Group 6	Change in NRS 0–10 (continuous)	ANOVA^a^
9 Distribution in *need for surgery*	Group 2 < Group 4 Group 4 > Group 6	Yes, don’t know, no	Summary statistics
Other outcomes			
10. Difference in *adherence to intervention* between groups	Group 2 > Group 4 Group 4 > Group 6 Group 2 > Group 6 We hypothesize differences between groups in adherence (%) to the training intervention (i.e., higher number of sessions per week, the lower adherence (%))	Number of sessions, sets, repetitions and time-under-tension	ANOVA^a^

^a^If data are not normally distributed the non-parametric Kruskal-Wallis test will be used

Analysis of variance: (ANOVA)

#### Primary analysis

**Fig. 6:**
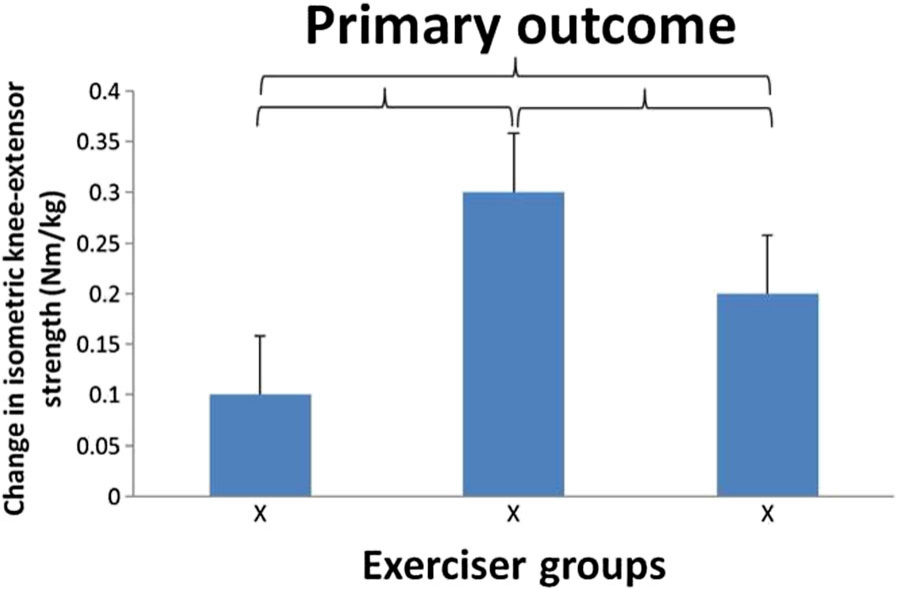
A hypothetical presentation of group changes from baseline to the primary endpoint (after 12 weeks of exercise) for the primary outcome, knee-extensor strength

The primary analysis will be between-group contrasts for the primary outcome at the primary endpoint. Figure 6 illustrates a hypothetical presentation of changes in isometric knee-extensor strength at the primary endpoint for the three groups.

#### Secondary analyses

**Fig. 7:**
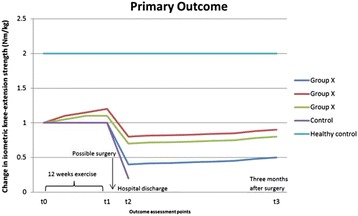
A hypothetical presentation of group changes in knee-extensor strength over the whole trial period. NB, control data are from the academic literature [11] and so are the healthy, age-matched, control data (age 66.8 years (6.5 SD)) [7]

The secondary analyses will be between-group contrasts for the secondary outcomes at the primary and secondary endpoints. Figure 7 illustrates a hypothetical presentation of changes in isometric knee-extensor strength for the three groups during the whole trial period.

For all outcomes (primary and secondary), mean scores with corresponding standard deviations (SD), and between-group contrasts (change scores) with corresponding 95% confidence intervals (95% CI) and *p* value, will be reported at each endpoint (t_0_, t_1_, t_2_ and t_3_) for each group (groups 2, 4 and 6) (Table 4).

**Table 4:** Outcomes for primary and secondary analyses

	t_0_ Baseline			t_1_ After 12-week exercise			t_2_ After surgery at hospital discharge			t_3_ 3 months after surgery			Between-group contrasts (change scores) 95% CI (*p*)
Mean, SD	Gp. 2	Gp. 4	Gp. 6	Gp. 2	Gp. 4	Gp. 6	Gp. 2	Gp. 4	Gp. 6	Gp. 2	Gp. 4	Gp. 6	
Isometric knee-extensor strength (Nm/kg)													Primary analysis Δ_t0-t1_ Secondary analysis Δ_t0- t2_, _t0-t3_
6-Minute Walking Test for distance (6MWT)													Secondary analysis Δ_t0-t1_, Δ_t0-t2_, Δ_t0-t3_
Stair Climb Test (SCT)													Secondary analysis Δ_t0-t1_, Δ_t0-t2_, Δ_t0-t3_
Knee Osteoarthritis Outcome Score (KOOS)							Na	Na	Na				Secondary analysis Δ_t0-t1_, Δ_t0-t3_
Oxford Knee Score (OKS)							Na	Na	Na				Secondary analysis Δ_t0-t1_, Δ_t0-t3_
Current knee pain (Numeric Rating Scale (NRS) 0–10)													Secondary analysis Δ_t0-t1_, Δ_t0-t2_, Δ_t0-t3_
Knee pain during the last week (NRS 0–10)							Na	Na	Na				Secondary analysis Δ_t0-t1_, Δ_t0-t3_
Need for surgery now (yes/don’t know/no)	Na	Na	Na				Na	Na	Na	Na	Na	Na	Secondary analysis
Exercise adherence													
• No. sessions (prescribed, completed, % completed)	Na	Na	Na							Na	Na	Na	Secondary analysis
• Seconds of total time-under-tension (TUT) (prescribed, completed, % completed)	Na	Na	Na							Na	Na	Na	Secondary analysis
Adverse events													Secondary analysis

The patients who choose not to be operated with TKA after the exercise period will be followed with annual outcome assessments as part of a small case study.

Descriptive statistics for the trial population will be presented as in Table 5.

**Table 5:** Descriptive statistics

	Gp. 2	Gp. 4	Gp. 6	All patients
Age (years)				
Height (cm)				
Weight (kg)				
Gender (m/f)				
Index knee (r/l)				
Kellgren-Lawrence classification (I–IV)				

#### Supplementary analyses

**Fig. 8:**
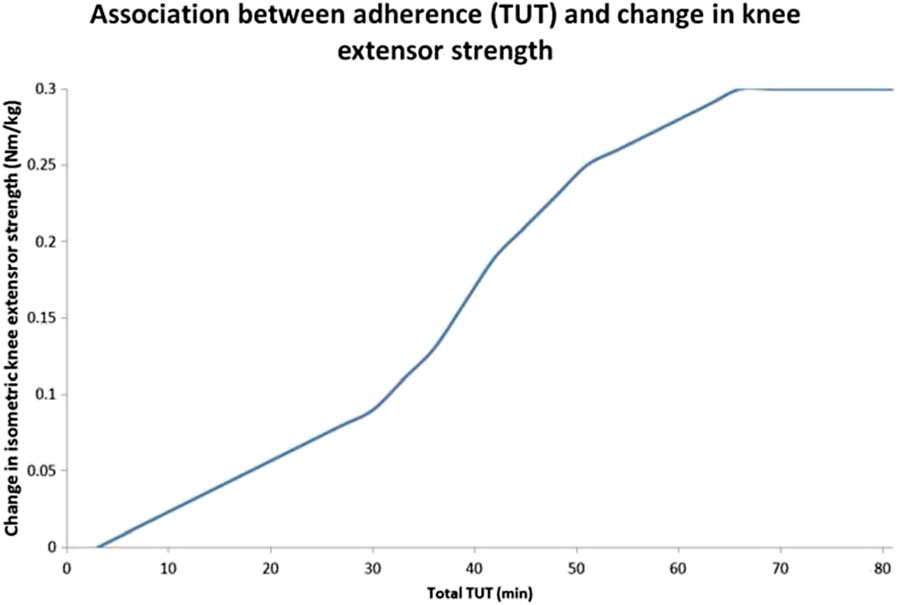
A hypothetical simple regression model with the change in isometric knee-extensor strength (Nm/kg) at the primary endpoint and time-under-tension (TUT)

The supplementary analyses will be simple regression models in which the three exercise dosage groups will be pooled, allowing us to utilize the full dataset; that is, the exercise dosage recorded by the sensors will be used in the analysis, not the prescribed exercise dosage. The dependent variables will be the change in primary and secondary outcomes from baseline to the primary endpoint and to the secondary endpoints. The independent variable will be exercise dosage, quantified in two ways: (1) as total TUT and (2) as number of completed exercise sessions for each patient (Table 6). Figure 8 illustrates a hypothetical simple regression model with the change in isometric knee-extensor strength (Nm/kg) at the primary endpoint and TUT.

**Table 6:** Regression models for supplementary analysis

Supplementary analyses (primary outcome at primary endpoint)		
	Dependent variable (y)	Independent variable (x)
Linear regression model	Change in *isometric knee-extensor strength* Δ_t0-t1_	Exercise adherence Seconds of total time-under-tension (TUT) Number of completed exercise sessions
Supplementary analyses (secondary outcomes at primary and secondary endpoints)		
Linear regression models	Change in *isometric knee-extensor strength* Δ_t0-t2_, Δ_t0-t3_	Exercise adherence Seconds of total time-under-tension (TUT) Number of completed exercise sessions
	Change in *6-minute walk test for distance* (6MWT) Δ_t0-t1, t0-t2, t0-t3_	
	Change in *stair climb test* (SCT) Δ_t0-t1, t0-t2, t0-t3_	
	Change in *Knee Osteoarthritis Outcome Score* (KOOS) subscales Δ _t0-t1, t0-t3_	
	Change in *Oxford Knee Score* (OKS) Δ _t0-t1, t0-t3_	
	Change in *current knee pain* (Numeric Rating Scale, NRS) Δ _t0-t1, t0-t2, t0-t3_	
	Change in *knee pain during the last week* (Numeric Rating Scale, NRS) Δ _t0-t1, t0-t3_	
	Need for surgery (yes/don’t know/no)	
	Averse events

**Fig. 9:**
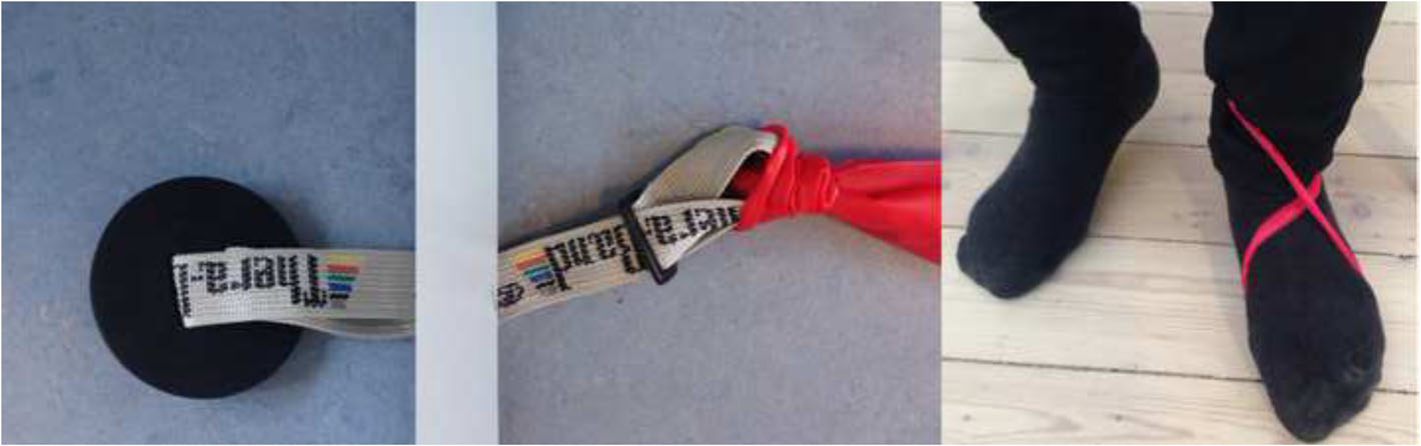
Elastic exercise band fixation to an immoveable object (e.g., an elastic exercise band anchor behind a closed door) and placement around the ankle of the exercise leg

### Missing data

All analyses will follow the intention-to-treat (ITT) principle with a clear registration and reporting of the drop-out rate. All patients will be analyzed as randomized. To create a full analysis dataset for the intention-to-treat analyses, missing data will be imputed using multiple imputations.

### Data monitoring

As the intervention(s) provided in the present trial poses little or no risk to the participating patients, no data monitoring committee will be composed. Funding sources of the current trial has no part in the design, conduction or reporting of the trial, thus there is no conflict of interests. As the intervention(s) poses little or no risk to the participating patients no interim analyses will be applied. Stopping guidelines for discontinuing and modifying the exercise has been described previously (see the “Criteria for modifying and discontinuing” section).

### Access to the final trial dataset

The principal investigator and principal supervisor will have full access to the dataset as well as all co-authors. A fully patient-anonymized dataset and corresponding statistical analysis code will be made available for the scientific journal reviewing the manuscript and its results within 6 months in line with the recent proposal from the International Committee of Medical Journal Editors (ICMJE) [52].

### Ancillary and post-trial care

The current trial is not planned to include patient ancillary care or post-trial care. On completion of the trial, if participants want to continue with the exercises independently they can do so. However, this will not be a part of the trial and will be on the patient’s own initiative.

### Dissemination policy

The QUADX-1 trial is planned to be reported in three manuscripts, which will be published in scientific peer-reviewed journals. All manuscripts will refer to the QUADX-1 trial’s Clinical.Trials.gov identifier. The first manuscript will be the trial protocol, the second manuscript will be the primary trial report of the investigated dose-response relationship, and the third manuscript will be a qualitative study investigating enablers and barriers for patient adherence to home-based exercise and physiotherapists’ experience in administering home-based exercise before TKA. The results will also be presented at relevant scientific conferences and symposiums. Trial participants will be informed of the trial via a letter explaining the results in layman’s terms. On request, the data underlying the results presented in the manuscript will be available to reproduce the findings. We intend to make the dataset – containing de-identified individual patient data – publicly available no later than 6 months after publication, consistent with the recent proposal from the International Committee of Medical Journal Editors (ICMJE) [52], if it complies with national regulations, e.g., the Danish Data Protection Agency. Trial data can be requested by contacting the main investigator (RSH) or trial director (TB). Positive as well as negative and inconclusive results will be published.

All contributors to the study will be offered to co-author the three above manuscripts if they fulfill the International Committee of Medical Journal Editors (ICMJE) recommendations for authorship [52]. No professional writers will be used.

## Discussion

In 2011, approximately 60,000 patients were registered in the Danish health care system with symptoms of knee OA and the annual incidence of knee OA has increased from 35.8 in 1997 to 155.2 in 2010 per 100,000 inhabitants [3]. Consequently, this population is very large and growing, which stresses the importance of optimizing the treatment offered to these patients. The *QUADX-1 trial* will add knowledge relating to which knee-extensor strength exercise dosage is most effective in increasing knee-extensor strength and whether a single, home-based (unsupervised) knee-extensor strength exercise is feasible in patients with end-stage knee OA.

The minimal treatment approach (single exercise) has been chosen as it is a pragmatic and time-saving solution [46]. Further, the rationale for investigating a home-based, single knee-extensor strength exercise is that it could improve exercise adherence as it is simple (minimal intellectual effort), does not take a long time (requires less surplus energy) and is likely to inflict less pain (less stress imposed on the knee joint). An exercise targeting the knee-extensor muscle is chosen as it is the single most important muscle for function in the knee OA population [9, 10, 13, 14].

In summary, the objective of the *QUADX-1 trial* is to investigate the efficacy of three different exercise dosages of pre-operative, home-based, knee-extensor strength exercise before and shortly after surgery in patients eligible for total knee replacement. The results will indicate which knee-extensor strength exercise dosage is most effective for increasing knee-extensor strength in the end-stage knee OA population. Furthermore, the results will indicate whether pre-operative knee-extensor strength exercise improves knee-extensor strength and function prior to surgery and whether this effect (if any) is sustained following surgery.

### Strengths

The trial design has several strengths as it addresses numerous gaps in the academic literature. Trials investigating the dose-response relationship of strength exercise in patients with end-stage knee OA are rare. Accordingly, there is a need for investigating the most effective exercise dose in this patient population, as highlighted in two recent systematic reviews using meta-analysis [21, 22]. Peer et al. highlight the scarcity of evidence related to exercise dose-response in patients with knee OA needed to guide pre-habilitation in clinical practice [22]. Further, Bartholdy et al. suggest that a 30–40% gain in knee-extensor strength is needed to positively affect pain and disability in patients with knee OA [21], highlighting the need for evidence to suggest the exercise dosage required to obtain adequate improvement in knee-extensor strength.

Adherence to home-based exercise is reported to be low with a high risk of over-reporting [45–48]. The use of sensors attached to the elastic exercise band will address this in an objective manner. Further, in a recent systematic review on adherence with physiotherapy exercises it was requested that the relationship between adherence and treatment outcome was investigated [46].

The exercise regimes currently offered to patients with knee OA are mostly supervised exercise sessions at outpatient clinics and comprise several exercises resulting in accumulated time spent and cost. In this trial, a single, simple, home-based exercise is applied, thus, investigating whether an alternative exercise treatment, which is simplified and maintained unsupervised at home, might be offered to these patients.

This trial protocol follows the Standard Protocol Items: Recommendations for Interventional Trials (SPIRIT) Checklist (Fig. 2, Additional file 1) [24] and the exercise intervention is reported according to the TIDieR Checklist (Additional file 2) [26] allowing for replication and direct clinical use, which has been requested in a recent review [53]. The cross-sectional design mimics everyday practice of cross-sector boarder communication increasing the external validity for future clinical implementation.

### Limitations

The trial has some limitations which must be taken into consideration. There is no control group limiting the inferences that can be made on the effect of the knee-extensor strength exercise.

Due to the nature of the intervention (exercise) there is a risk of selection bias, e.g., patients motivated for exercise are more likely to participate in the trial. This also limits the external validity with respect the whole knee OA population.

In the present trial design, the patients are asked about their *need for surgery* after the 12-week exercise period. In line with the above limitation, the answer to this question could be biased as patients motivated for exercise might be less motivated towards surgery affecting their answer to *need for surgery* towards.

Though the sensor objectively measures activity with the elastic exercise band, the sensor cannot measure who is exercising, which muscle is exercised or what movement is performed.

Finally, no recording or monitoring of the use of knee-related pain medication during the trial is planned for.

## Trial status

Protocol version no. 4.1 (21 November 2017). Inclusion began 1 November 2016. Approximate date for inclusion completion is 31 December 2019.

## Additional files

Additional file 1: SPIRIT Checklist. (DOC 122 kb)

Additional file 2: TIDieR Checklist. (DOCX 31 kb)

Additional file 3: Patient brochure (English). (PDF 825 kb)

Additional file 4: Administrative information. (DOCX 38 kb)

## Abbreviations

6MWT: 6-Minute Walking Test
ASA: American Society of Anesthesiologists’ physical status classification
ICC: Intraclass correlation coefficient
ITT: Intention-to-treat
KOOS: Knee Osteoarthritis Outcome Score
MDC: Minimal detectable change
Nm/kg: Newton-meter (torque) per kilogram body mass
NRS: Numeric Rating Scale
OA: Osteoarthritis
OKS: Oxford Knee Score
RM: Repetitions-maximum
SCT: Stair Climb Test
SEM: Standard error of measurement
TKA: Total knee arthroplasty
TUT: Time-under-tension
