# Supplementary material for: Efficacy of pre-operative quadriceps strength training on knee-extensor strength before and shortly following total knee arthroplasty: protocol for a randomized, dose-response trial (The QUADX-1 trial)
Source: Trials. 2018 Jan 18;19:47. doi: 10.1186/s13063-017-2366-9 (PMC5774158; doi:10.1186/s13063-017-2366-9)
Supplement: Supplementary file 3 — Patient brochure (English). (PDF 825 kb) [file 13063_2017_2366_MOESM3_ESM.pdf]

Exercise instruction

Attachment of elastic exercise band

The elastic exercise band is mounted on an anchor. The anchor is placed under a door and the door is closed (Figure 1).

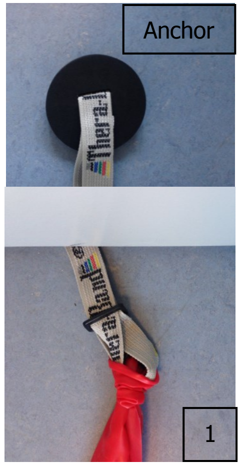

Placement of chair

The elastic exercise band is placed on the floor extending out from the door and a chair is placed above the elastic exercise band (Figure 2).

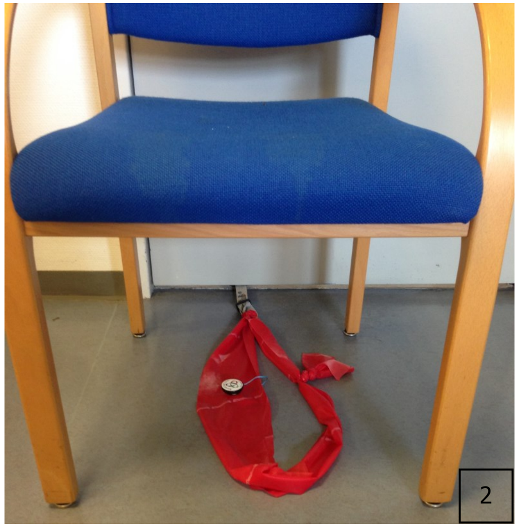

Attaching elastic exercise band to the ankle

Sitting in the chair attach the elastic exercise band to the ankle as shown in Figure 3-6.

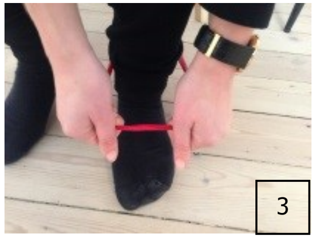

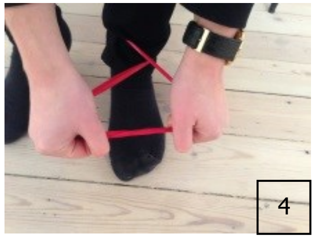

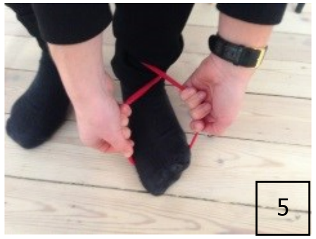

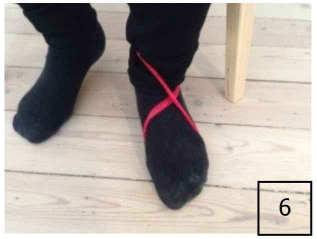

Position in chair

Place a pillow under your thigh of the exercising leg so that your foot is just in contact with the floor (Figure 7). Now move the chair away from the door to increase the resistance in the elastic exercise band. You are now ready to begin the knee-extensor exercise.

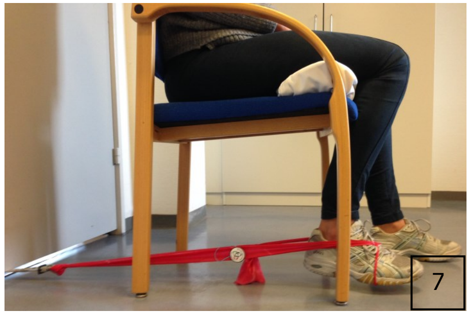

Knee-extensor exercise

Starting position: The exercise leg is flexed 90 degrees with the foot resting on the floor (Figure 7). Now you *extend* your knee slowly (**3 seconds**) until fully extended (Figure 8). Keep your knee *extended* for **1 second** and then *flex* it slowly (**4 seconds**) until your knee is back in the starting position with the foot resting on the floor.

The exercise is performed in 3 sets with 12 repetitions in each set. The resistance in the elastic exercise band must be very high, meaning that you are only just able to complete the 12 repetitions. At the 12th repetition you should feel *fatigued* in your thigh muscles. If you can complete more than 12 repetitions the resistance in the elastic exercise band has to be increased. You increase the resistance in the elastic exercise band by moving the chair away from the door.

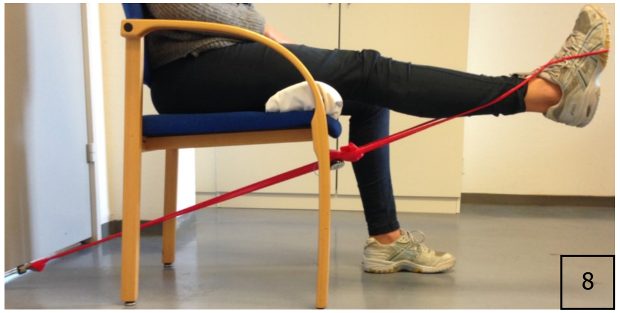

Purpose

The purpose of the knee-extensor exercise is to improve strength in the muscles on the front of your thigh, reduce knee pain and symptoms, and provide and better basis for deciding further treatment cf. recommendations from the National Board of Health.

Project overview

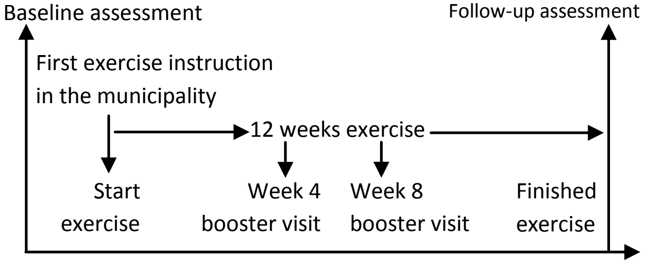

Exercise frequency

You should exercise 2/4/6 (NB. Depending on randomization) times a week according to the exercise instruction describe in this brochure.

Expected effect

It can take up to 12 weeks of exercising before you experience improvement in your knee symptoms. Hence, it is very important that you keep exercising even though you do not experience improvement right away.

Knee symptoms while exercising

Knee pain during exercising is allowed as long the pain is tolerable. There is no risk associated with this. If you experience too much knee pain you should lessen the resistance in the elastic exercise band by moving the chair closer to the door. If you experience unacceptable knee pain you should stop exercising until your knee pain is decreased sufficiently to exercise again. Muscle soreness in the thigh can occur. There is no risk associated with this and the soreness will go away within 2-3 days.

Contact

For more information on the exercise, potential symptoms or the project in general contact:

Primary investigator, physiotherapist
Rasmus Skov Husted
E-mail: rasmus.skov.husted@regionh.dk
Telefon: 29 78 56 68

Benefits, disadvantages and risks

At present we do not know what benefits might come from completing the exercise as the purpose of the project is to investigate this. No disadvantages or risks are expected to be associated with completing the exercise.

This brochure is designed by Rasmus Skov Husted, physiotherapist and primary investigator at the Clinical Research Centre, University Hospital Hvidovre. Last revision January 2017.

Exercise against osteoarthritis of the knee

Region H Hvidovre Hospital

Københavns Kommune
