# Supplementary material for: Efficacy of pre-operative quadriceps strength training on knee-extensor strength before and shortly following total knee arthroplasty: protocol for a randomized, dose-response trial (The QUADX-1 trial)
Source: Trials. 2018 Jan 18;19:47. doi: 10.1186/s13063-017-2366-9 (PMC5774158; doi:10.1186/s13063-017-2366-9)
Supplement: Supplementary file 4 — Administrative information. (DOCX 38 kb) [file 13063_2017_2366_MOESM4_ESM.docx]

## Administrative information

### Title

Efficacy of pre-operative quadriceps strength training on knee-extensor strength before and shortly following total knee arthroplasty: Protocol for a randomized, dose-response trial (The QUADX-1 trial)

Trial registration
The trial was pre-registered at ClinicalTrials.gov 10/10-2016, Id: NCT02931058, <https://clinicaltrials.gov/ct2/show/NCT02931058>.

### Items from the World Health Organization Trial Registration Data Set

| **WHO Trial Registration Data Set (Version 1.2.1)** | |
| --- | --- |
| Item | Information |
| 1 - Primary Registry and Trial Identifying Number | Ethical Committee of the Capital Region Denmark:  H-16025136.  Danish Data Protection Agency:  J. nr.: 2012-58-0004  Lokale RegH j. nr.: AHH-2016-072, med I-Suite nr.: 04980  ClinicalTrials.gov:  Identifier: NCT02931058  <https://clinicaltrials.gov/ct2/show/NCT02931058> |
| 2 - Date of Registration in Primary Registry | Ethical Committee of the Capital Region Denmark: Confirmation received 13/9/2016.  Danish Data Protection Agency:  Confirmation received 15/9/2016.  ClinicalTrials.gov:  10/10-2016 |
| 3 - Secondary Identifying Numbers |  |
| 4 - Source(s) of Monetary or Material Support | The Capital Region´s strategic funds (1.500.000 dkkr) and the Capital Region´s fund for cross-continuum research (500.000 dkkr). |
| 5 - Primary Sponsor | Physical Medicine & Rehabilitation Research – Copenhagen group (PMR-C), the Clinical Research Centre, and Clinical Orthopaedic Research Hvidovre (CORH), Hvidovre Hospital, University of Copenhagen, Copenhagen, Denmark. |
| 6 - Secondary Sponsor(s) | None. |
| 7 - Contact for Public Queries | RSH ([rasmus.skov.husted@regionh.dk](mailto:rasmus.skov.husted@regionh.dk))  TB ([thomas.quaade.bandholm@regionh.dk](mailto:thomas.quaade.bandholm@regionh.dk)) |
| 8 - Contact for Scientific Queries | Principal Investigator(s)  Rasmus Skov Husted (RSH)  Physiotherapist, M.Sc.  PhD student  Clinical Research Center (Section 056) Hvidovre Hospital, University of Copenhagen  Kettegaard Allé 30 DK-2650 Hvidovre, Denmark  Phone: +45 38626035 - Mobil: +45 20958645  E-mail: [rasmus.skov.husted@regionh.dk](mailto:rasmus.skov.husted@regionh.dk)  Thomas Bandholm, PhD Professor, Head of Research  Physical Medicine & Rehabilitation Research - Copenhagen (PMR-C) Clinical Research Center (Section 056), Hvidovre Hospital, University of Copenhagen, Kettegaard Allé 30 DK-2650 Hvidovre, Denmark.  Phone: +45 38626344  E-mail: [thomas.quaade.bandholm@regionh.dk](mailto:thomas.quaade.bandholm@regionh.dk) |
| 9 - Public Title | Quadriceps exercise before total knee arthroplasty (The QUADX-1 trial) |
| 10 - Scientific Title | Efficacy of pre-operative quadriceps strength training on knee-extensor strength before and shortly following total knee arthroplasty: A randomized dose-response trial (The QUADX-1 Trial). |
| 11 - Countries of Recruitment | The municipalities of Copenhagen, Hvidovre and Brøndby, Denmark. |
| 12 - Health Condition(s) or Problem(s) Studied | Patients with end-stage knee osteoarthritis |
| 13 - Intervention(s) | Home-based knee-extensor exercise (3 sets of 12 repetitions maximum) 2, 4 or 6 times a week for 12 weeks. The exercise will be performed with an elastic exercise band with an attached sensor registering when the patients exercise, how much they exercise and how they exercise. |
| 14 - Key Inclusion and Exclusion Criteria | Inclusion criteria   - Patient is a possible candidate for a primary TKA due to end-stage knee osteoarthritis, based on the below terms:   - Knee pain >3 (Numeric Rating Scale) in the last week   - Kellgren-Lawrence classification grade ≥2 - Patient is eligible for home-based knee-extensor exercise - Patient has an age ≥ 45 years - Patient is resident in one of the three municipalities (København, Hvidovre or Brøndby) involved in the trial - Patient is able to speak and understand Danish   Exclusion criteria   - Exercise is contra-indicated for the patient - Patient has a neurological disorder - Patient has a diagnosed systemic disease (ASA score ≥ 4) - Patients with terminal illness - Patient has severe bone deformity demanding use of nonstandard implants - Weekly alcohol consumption above national recommendations (>7 units women, >14 units men) |
| 15 - Study Type | The trial will use a three arm, parallel-group prospective randomized trial study design, using consecutive sampling of patients. The included patients will be randomized to one of three dosages of pre-operative knee-extensor exercise and assessed blinded pre-exercise (baseline), after 12 weeks of exercise (before possible surgery), at hospital discharge (3-8 days post-surgery) and three months after surgery. |
| 16 - Date of First Enrollment | November 2016 |
| 17 - Target Sample Size | The trial will enroll 3x42 = 126 patients (42 pr. exercise group). To account for a 10% drop-out 140 patients will be included in total (3 x 42 + 14 = 140). |
| 18 - Recruitment Status | - - **Recruiting:** participants are currently being recruited and enrolled |
| 19 - Primary Outcome(s) | Isometric knee-extensor strength at 60 degrees knee flexion (Nm/kg body mass).  The time point of primary interest is after 12 weeks of exercise. Secondary time points of interest are just before hospital discharge after surgery and three months after surgery. |
| 20 - Key Secondary Outcomes | Performance-based function (walking distance in 6 minutes and climbing of stairs), knee pain (numerical rating scale), self-reported disability (Knee Osteoarthritis Outcome Score and Oxford Knee Score), need for surgery and exercise adherence (sessions) and volume (sets, reps, time under tension).  The time point of primary interest is after 12 weeks of exercise. Secondary time points of interest are just before hospital discharge after surgery and three months after surgery. |

Protocol version
Version 4.1 (21/11-2017)
Authors: RSH, MSR, KT, AT, HH and TB

Funding
The Capital Region´s strategic funds (1.500.000 dkkr (financial)) and the Capital Region´s fund for cross-continuum research (500.000 dkkr (financial)).

### Roles and responsibilities

The trial design, daily operation and completion is conducted in collaboration between Physical Medicine & Rehabilitation Research – Copenhagen (PMR-C), Clinical Orthopedic Research Hvidovre (CORH), both located at Copenhagen University Hospital, Hvidovre, Copenhagen, Denmark.

Rasmus Skov Husted (RSH)^1,2,3^
Anders Troelsen (AT)^3^
Kristian Thorborg (KT)^1,4^
Michael Rathleff (MR)^5,6^
Henrik Husted (HH)^3^
Thomas Bandholm (TB)^1,2^

^1^Physical Medicine & Rehabilitation Research-Copenhagen (PMR-C); Department of Physical and Occupational Therapy, and Clinical Research Centre, Hvidovre Hospital, University of Copenhagen, Hvidovre, Denmark

^2^Optimed, Clinical Research Centre, Copenhagen University Hospital, Amager-Hvidovre, Kettegård Alle 30, 2650 Hvidovre, Denmark

^3^Clinical Orthopedic Research Hvidovre (CORH), Department of Orthopedic Surgery, University Hospital of Hvidovre, Copenhagen, Denmark

^4^Sports Orthopaedic Research Center – Copenhagen (SORC-C), Arthroscopic Center Amager, Department of Orthopedic Surgery, Copenhagen University Hospital, Copenhagen, Amager-Hvidovre, Denmark

^5^Research Unit for General Practice in Aalborg, Department of Clinical Medicine, Aalborg University, Denmark

^6^Department of Occupational Therapy and Physiotherapy, Aalborg University Hospital, Aalborg, Denmark

TB drafted the original idea for the trial and initiated the trial design. AT, KT, MSR, HH, and RSH later contributed to the trial design process. TB and RSH drafted the trial protocol and all other authors contributed and approved the final version of the protocol. TB is the main grant holder and responsible for the completion of the trial. RSH is the trial manager and principal investigator being responsible for daily operation, coordination between trial collaborators, outcome assessment of patients, collection and structuring of data, ensuring compliance with milestones and completion of the trial. RSH will help train the trial physiotherapists in the use of the elastic exercise band and sensor. RSH will draft the manuscripts for publication with contribution and approval of the final version from all co-authors.

Name and contact information for the trial sponsor
Physical Medicine & Rehabilitation Research-Copenhagen (PMR-C); Department of Physical and Occupational Therapy, and Clinical Research Centre Hvidovre Hospital, University of Copenhagen, Hvidovre, Denmark. Contact: Rasmus Skov Husted ([rasmus.skov.husted@regionh.dk](mailto:rasmus.skov.husted@regionh.dk)) and Thomas Bandholm ([thomas.quaade.bandholm@regionh.dk](mailto:thomas.quaade.bandholm@regionh.dk))

Clinical Orthopedic Research Hvidovre (CORH), Department of Orthopedic Surgery, University Hospital of Hvidovre, Copenhagen, Denmark. Contact: Anders Troelsen ([anders.troelsen@regionh.dk](mailto:anders.troelsen@regionh.dk))

The trial is independent of the sponsors and funders. That is, the funders had no part in trial design, data collection, trial management, analysis or interpretation of data. Further, the funders had no part in writing the report or manuscripts for publication and will have no part in the decision to submit the report and manuscripts for publication, thus the funders have no authority over any of these activities.

| Contributors | Roles responsibilities |
| --- | --- |
| Primary investigator (RSH) | - Designing of the QUADX-1 trial - Coordination between all involved parts (internal (Hvidovre Hospital) and external (involved municipalities)) - All tasks involving the daily operation of the trial - Organizing steering committee and working group meetings - Drafting information papers (e.g. exercise instructions) for patients and trial personnel - Instructing trial physiotherapists in the use of the elastic exercise band (BandCizer) - Drafting manuscripts for publication |
| Primary supervisor (TB) | - Overall responsibility for the trial - Designing the QUADX-1 trial - Member of the Steering Committee - Member of the Data Management Team |
| Steering Committee Team (RSH, TB, AT, HH, MR, KT, and employees from the collaborating municipalities) | - Overseeing the progress of the trial - Contributing to solutions, should the original trial plan not be applicable |
| Working Group (same members as the steering committee plus the trial physiotherapists) | - Sparring and coordination of the daily operation of the trial out in the involved municipalities |
| Data Management Team (RSH, TB, AT, HH, MR, KT) | - Planning of analyses - Handling of data |
